# Describing the physiological responses of different rice genotypes to salt stress using sigmoid and piecewise linear functions

**DOI:** 10.1016/j.fcr.2017.05.001

**Published:** 2018-05-01

**Authors:** Ando M. Radanielson, Olivyn Angeles, Tao Li, Abdelbagi M. Ismail, Donald S. Gaydon

**Affiliations:** a International Rice Research Institute, Crop and Environmental Sciences Division DAPO BOX 7777, Metro Manila, Philippines; b CSIRO Agriculture and Food, Brisbane, Australia; c School of Agriculture and Food Sciences, University of Queensland, St Lucia, Brisbane, Australia

**Keywords:** Leaf photosynthesis, Modelling, Rice, Salt-stress, Water-stress

## Abstract

Rice is the staple food for almost half of the world population. In South and South East Asia, about 40% of rice production is from deltaic regions that are vulnerable to salt stress. A quantitative approach was developed for characterizing genotypic variability in biomass production, leaf transpiration rate and leaf net photosynthesis responses to salinity during the vegetative stage, with the aim of developing efficient screening protocols to accelerate breeding varieties adapted to salt-affected areas. Three varieties were evaluated in pots under greenhouse conditions and in the field, with average soil salinity ranging from 2 to 12 dS m^−1^. Plant biomass, net photosynthesis rate, leaf transpiration rate and leaf conductance were measured at regular intervals. Crop responses were fitted using a logistic function with three parameters: 1) maximum rate under control conditions (Y_max_), 2) salinity level for 50% of reduction (b), and 3) rate of reduction (*a)*. Variation in the three parameters correlated significantly with variation in plant biomass production under increasing salinity. Salt stress levels that caused 50% reduction in net leaf photosynthesis and transpiration rates were higher in the tolerant genotype BRRI Dhan47 (16.5 dS m^−1^ and 14.3 dS m^−1^, respectively) than the sensitive genotype IR29 (11.1 dS m^−1^ and 6.8 dS m^−1^). In BRRI Dhan47, the threshold beyond which growth was significantly reduced was above 5 dS m^−1^ and the rate of growth reduction beyond this threshold was as low as 4% per unit increase in salinity. This quantitative approach to screening for salinity tolerance in rice offers a means to better understand rice growth under salt stress and, using simulation modelling, can provide an improved tool for varietal characterization.

## Highlights

• Rice crop responses to soil salinity can be fitted with a logistic function with three parameters.
• Tolerant genotype, BRRI Dhan 47 presented 50% of reduction in leaf net photosynthesis and transpiration at soil salinity higher than 14 dS m^−1^.
• Growth of tolerant genotype as BRRI Dhan 47 was significantly reduced at soil salinity higher than 5 dS m^−1^.

## 1 Introduction

Salinity is one of the main limiting environmental factors for crop production worldwide. Its occurrence and severity are expected to increase by around 25% by 2050 in vulnerable regions, particularly in deltaic costal zones (Dasgupta et al., 2014) where rice growing areas account for more than 65% of global production, making salinity one of the major threats to food security. Rice has been classified as salt-sensitive (Maas and Hoffman, 1977). Salinity has different effects on yield and growth, depending on crop stage, stress severity and duration, as well as the tolerance of the variety (Lutts et al., 1995, Zeng and Shannon, 2000). Rice yield has been estimated to decrease by 50% at a salinity level of 6.9 dS m^−1^ (Grattan et al., 2002). Addressing salt stress by developing improved salt-tolerant rice cultivars could mitigate the effects of salinity on rice production and contribute to improving food security at a global scale.

Salinity tolerance in rice results from complex interactions between environmental and genetic factors. Numerous studies have been conducted to understand salt stress tolerance mechanisms such as minimizing sodium (Na^+^) uptake and excluding Na^+^ from the shoot (Munns and Tester, 2008, Platten et al., 2013, Ismail and Horie, 2017). Candidate genes have been identified that confer salinity tolerance through regulating ion transport, osmoprotection and growth acceleration (Munns and Tester, 2008, Horie et al., 2012, Ismail and Horie, 2017). This understanding has contributed significantly to the successful breeding of salt-tolerant varieties and for identifying suitable donors for breeding programs (Garg et al., 2002, Islam et al., 2008). However, quantifying the contribution of these mechanisms at the cellular and molecular level in relation to their functions at the whole plant scale and under field conditions still remains unresolved.

Maas and Hoffman (1977) described plant responses to salinity using a simple quadratic equation, which defines the rate of yield decrease per unit of salinity increase, and the critical level of salinity at which a significant decrease in yield was observed. Thus far, these parameters have not been correlated with physiological processes occurring in the plant. Considering that yield results from the combination of numerous complex processes, the use of this equation may not be suitable for screening for salinity tolerance traits. Understanding salinity response processes at different scales within the plant could play an important role in accelerating progress for developing salt-tolerant high yielding rice varieties (Horie et al., 2012). Ion and nutrient imbalances, adjustment of water status, stomatal conductance, and reduction in photosynthetic activity are among the physiological responses occurring at the plant level (Horie et al., 2012). These responses have been reported to follow a two-step process with an initial water deficit effect over a short term and an ion toxicity effect over a longer term (Munns et al., 1995). Specific responses to salt stress involve mechanisms limiting salt entry and excluding salt at the whole plant and/or cellular level, especially from active tissues such as young leaves and reproductive parts. This mechanism preserves the photosynthetic apparatus and other physiological processes necessary for plant survival and growth. Salinity also has a direct effect on cell expansion and division, as typically reflected by a reduction in leaf area (Matthews et al., 1984, Netondo et al., 2004). Genotypic variability in the concentration of Na^+^ accumulating in plant tissue under salt stress was reported to be associated with variation in photosynthesis (Moradi and Ismail, 2007). This can even be observed at salinity levels lower than 2 dS m^−1^, at which symptoms of the effects of salinity are not yet visible (Yeo, 1998).

In addition to variations in salt exclusion and salt partitioning to roots and old tissues, variability in responses of photosynthetic traits to salinity is also important − largely the result of variability in tissue tolerance of accumulated salts. Tolerance to salinity during the vegetative and reproductive stages of rice involves numerous different mechanisms that contribute to the whole plant growth and productivity (Moradi et al., 2003, Singh and Flowers, 2010). Exposure of rice to salinity around the time of panicle initiation and flowering affects the growth and development of panicles and spikelets causing spikelet sterility, thus limiting sink size and grain formation. Senescence of expanded leaves is also accelerated by salinity, partially reducing assimilation due to the reduction of the photosynthetically active leaf area and further reducing assimilates allocated to grains. These processes result in changing source-sink relationships within the crop, leading to a reduction in grain yield.

Quantifying the variability of these physiological mechanisms using mathematical equations provides a means to describe and disaggregate the contribution of different limiting factors, particularly the genotypic component of the variability in crop responses. This approach could be used to develop trait-based modelling − a framework to test hypotheses about crop performance and to integrate the complex unpredictable variability of soil salinity in the field, as one of the main limitations in developing adapted varieties and technologies to overcome the effects of salinity (Skaggs et al., 2006, Crescimanno and Garofalo, 2006, Soltani and Sinclair, 2012).

In this study, a mathematical equation was developed to quantify effects of salinity on rice crops during the vegetative stage. Three genotypes with contrasting salinity tolerance were evaluated in both greenhouse and field experiments. Plant growth and leaf gas exchange, in response to four different levels of salinity treatment during the vegetative stage, were assessed. The patterns of these responses were used to develop an equation incorporating traits and varietal differences related to salt stress.

## 2 Materials and methods

### 2.1 Plant materials and growth conditions

Rice response to salinity treatment during vegetative stage was characterized over a series of four experiments (Table 1). Genotypic variability in growth responses to salinity was evaluated in three greenhouse experiments (Experiment 1, 2 and 3). A field experiment was used to evaluate responses at the plant population (crop) level and to validate genotypic variability and salinity effects observed in the greenhouse experiments. The greenhouse experiments were conducted at the International Rice Research Institute (IRRI) Los Baños, Philippines (14.17 N, 121.26 E), using three rice genotypes (Table 1); IR29, IR64 and BRRI Dhan47, characterized as sensitive, intermediate and tolerant of salt stress, respectively (Moradi et al., 2003, Islam et al., 2008). The genotypes were grown under four salinity levels, with electrical conductivities (EC) of 2 dS m^−1^, 4 dS m^−1^, 8 dS m^−1^ and 12 dS m^−1^, which were achieved by adding 0, 20, 55 and 90 mM of NaCl, respectively, to the nutrient solution (Supplementary Table 1).

**Table 1:** Summary of the four experiments conducted in this study, including genotypes used, salinity treatments, dates of sowing and start and termination of salt stress treatments. DAS: Days after sowing; Control: non-stressed conditions; T1, T2, T3 Treatments with approximately 20, 55 and 90 mM of NaCl applied to maintain solution electrical conductivities equivalent to 4, 8 and 12 dS m^−1^, respectively.

Experiments	Sowing date	Start of stress DAS	End of stress DAS	Genotypes	Location (Philippines)	Salinity treatments
Expt. 1	June 13, 2012	21	42	IR29, IR64, BRRI Dhan47	IRRI Experiment station, Los Baños	Control, T1, T2, T3
Expt. 2	February 11, 2013	20	47	IR29, IR64, BRRI Dhan47	IRRI Experiment station, Los Baños	Control, T1, T2, T3
Expt. 3	May 20, 2013	18	41	IR29, BRRI Dhan47	IRRI Experiment station Los Baños	Control, T2
Expt. 4	January 26, 2013	19	113	IR29, IR64, BRRI Dhan47	On farm experiment Infanta, Quezon city	Control, T2

Seeds were sown in polyvinyl chloride (PVC) pots filled with about 2.5 kg of gravel (20 cm depth and 20 cm of diameter) in Experiments 1, 2 and 3. The three experiments were arranged in randomized block design with salinity treatment as main factor and genotypes as the subplot factor. The pots were submerged in tanks filled with a nutrient solution. Each tank contained 96 pots arranged to maintain 20 cm spacing between plants. Four tanks corresponding to the four salinity treatments were used in Experiments 1 and 2. Two tanks were used (control and Treatment 2) in Experiment 3. Each genotype was represented by 32 pots in each tank with one plant per pot, with pots rotated weekly when the nutrient solution was renewed. The pH of the nutrient solution in each tank was adjusted daily to 5.5, and salinity was recorded hourly using a 5TE sensor connected to an EM50 datalogger (Decagon Devices, Inc. USA, Supplementary Fig. 1) at 10 cm depth. Plants were grown in a nutrient solution from sowing until the start of the salinity treatment, then exposed to their respective salinity treatments from 21 to 50 d after sowing (DAS) in Treatments 1 and 2. For Treatment 3 (12 dS m^−1^), the treatment was terminated at 35 DAS (when most of the fully expanded leaves of the sensitive genotype, IR29, senesced) to permit recovery before flowering, after which plants were maintained using the control treatment solution. The climatic conditions inside the greenhouse were monitored daily by an automated weather station (Davis Instruments, USA) recording radiation, air temperature and air relative humidity (Table 2). The air temperature was partially controlled via ceiling ventilation in the greenhouse and the relative humidity varied upwards from a minimum of 70%.

**Table 2:** Environmental conditions in Experiment 1, 2, 3 and 4. T_mean_: average daily mean temperature during the crop growth period; T_max_: average maximum daily temperature; RH: mean air relative humidity; PAR: average photosynthetically active fraction of total radiation; VPD: vapour pressure deficit. Data are means ± SE of daily values between period from sowing to 50 days after sowing and from 50 to 130 days after sowing in each experiment.

Variables	Experiment 1		Experiment 2		Experiment 3		Experiment 4	
	0-50 DAS	51 −130 DAS	0-50 DAS	51 −130 DAS	0-50 DAS	51 −130 DAS	0-50 DAS	51–130 DAS
T_mean_ (^°^C ± sd)	31 ± 2	30 ± 2	24 ± 1	25 ± 1	25 ± 1	25 ± 1	26 ± 1	29 ± 1
T_max_ (^°^C ± sd)	37 ± 4	34 ± 3	33 ± 3	35 ± 2	33 ± 3	36 ± 2	29 ± 2	33 ± 1
RH (% ± sd)	76 ± 4	79 ± 5	79 ± 3	79 ± 1	78 ± 3	78 ± 1	86.1 ± 4.7	80.4 ± 4.2
PAR (mol m^−2^ d^−1^ ± sd)	6.70 ± 2.25	5.82 ± 2.14	6.88 ± 1.8	6.7 ± 1.0	5.98 ± 1.8	7.15 ± 1.2	6.14 ± 1.7	7.38 ± 1.3
VPD (kPa ± sd)	1.07 ± 0.26	0.89 ± 0.27	0.83 ± 0.17	0.90 ± 0.07	0.83 ± 0.17	0.86 ± 0.08	0.56 ± 0.0	0.53 ± 0.0
Number of days Tmax > 35^ °^C	37	28	7	66	29	14	0	7

The on-farm field experiment (Experiment 4) was conducted in Infanta, Philippines (14.75 N, 121.68 E) from 26 January to 21 May 2013 during the dry season. The three genotypes were grown in the field using a split-plot design with salinity treatment as the main plot and genotype as sub-plot factors. Seedlings were transplanted at 21 DAS with a density of 2 plants hill^−1^ and 25 hills m^−2^.

The field was managed to limit weed, pest and disease infestation. Fertilizers were applied at rates of 220:40:40 kg ha^−1^ N: P_2_O_5_: K_2_O, with N applied in 3 splits (basal, 36 and 44 DAS). The ponded water in the field was maintained at 3–5 cm throughout the season. Two salinity treatments were applied; 1) a non-stressed (NS) control treatment continuously irrigated with fresh ground water and, 2) a salt stress treatment irrigated with saline river canal water (SW) from five days after transplanting to harvest. To achieve salinity levels higher than 8 dS m^−1^ at 15 cm soil depth in SW plots, about 10 kg of sea salt was broadcasted in the ponded water in each treated plot. Salinity of the soil solution was hourly recorded using a 5TE sensor connected to an EM50 datalogger (Decagon Devices, Inc. USA, Supplementary Fig. 2), placed at 10 cm depth in each main plot for each salinity treatment. Soil salinity reached an average seasonal value of 7.2 dS m^−1^ (with a maximum of 14.97 dS m^−1^) in the SW plots and around 0.96 dS m^−1^ in the NS plots (Supplementary Fig. 2).

### 2.2 Plant growth and biomass measurements

Time to panicle initiation and flowering (DAS) for 4–6 plants per salinity treatment and genotype in Experiments 1 and 2 were recorded according to the standard description of rice phenology (Counce et al., 2000). Data were converted into accumulated thermal time from sowing (using 8 °C as base temperature) and then averaged to determine panicle initiation and flowering time for each genotype under each salinity treatment. Plant samples were collected before, during (28, 35, 42 DAS), at the end of the stress period, and at flowering. Plant height, tiller number, leaf area and biomass were measured using three to six plants per genotype and per treatment at each sampling time. Plants were then separated into green leaves, dead leaves, stems and roots. Green leaf area was measured using a Licor leaf area meter (Li-COR Inc., Lincoln, NE, USA). Each plant component was then oven dried at 70 ^°^C to a constant weight to determine its dry biomass.

### 2.3 Leaf gas exchange measurements

Three weeks after the start of the treatments, net leaf photosynthesis, leaf conductance and transpiration rate were measured three times at 7 day interval, from the start of salinity treatment (28, 35, 42 DAS) on four to six plants per genotype per treatment, in the greenhouse experiments. In the field, for each salinity treatment and each genotype, gas exchange measurements were performed once on 6 hills per replicate at 42 DAS. Measurements were taken with a Licor 6400 (LI-COR Inc., Lincoln, NE, USA) on the youngest four fully expanded leaves of the main tiller. The last two expanded leaves were defined as younger leaves and the other two preceding based on their rank along the main stem were defined as older leaves. Leaf net photosynthesis is defined as the net assimilation rate of carbon dioxide per leaf area (μmol CO_2_ m^−2^ s^−1^) and leaf transpiration rate is defined as the amount of water transpired per unit of leaf area (mmol H_2_O m^−2^ s^−1^). Measurements were taken between 10 a.m. and 2 p.m. The leaf chamber conditions were set at a mean temperature of 30 ^°^C and relative humidity of 60%. Light was set at PAR of 1000 mmol m^−2^ s^−1^.

### 2.4 Data analysis

Statistical analysis including piecewise linear and logistic function-fitting was performed using R software (R Development Core Team, 2008). Student T-tests and pairwise multiple parameter comparisons were used to compare means and to determine significance groups (Sheskin, 2003). Variance analysis was performed to assess the effects of genotype and salinity treatment, experiment and time of measurement as number of days after sowing; and the interaction of genotype x salinity treatment. The mean square of the sum of errors (MSE) was used to quantify the weight of the contribution of a factor to the variability of a variable, and the *P-*value was used to assess the significance of the factor effect at a 95% confidence level (*P *≤ 0.05). As variabilities within experiments were observed for plant growth and salinity effects, comparison of genotype responses to salinity was performed using total plant biomass (kg ha^−1^), leaf area index (m^2^ m^−2^), plant height (cm) and tiller number, leaf net photosynthesis rate, transpiration rate and leaf conductance under salinity treatments relative to average values for respective genotypes grown under control conditions. Absolute values for plants grown under control conditions were recorded per plant (Experiment 1, 2 and 3) and per area of sampling of the 6 hills (Experiment 4). Relative values were calculated as the ratio of the values measured under salinity treatment to average values of the control and are reported as unitless values. Plant biomass was reported in kg ha^−1^ assuming a plant density of 25 plants m^−2^ at 20 cm × 20 cm spacing. Plant growth measurements at flowering time were used to evaluate the ability of the genotype to recover after the salinity treatment during the vegetative stage in Experiment 1 and 2.

Leaf gas exchange parameters in response to salinity were fitted with a two piecewise linear function and a logistic function using the Segmented and the LME packages of the R software.

The two piecewise linear function used three parameters as follows:

(1) $$\begin{array}{c} \text{ If x}<{\text{EC}}_{\text{c}\text{r}}\\ \begin{array}{c} \begin{array}{c} \;\;\;\;\;\;\;\;\;\;\;\;\;\;\;\;\;\;\;\;\;\;\;\;\;y=a_{1}x+\beta_{1} \end{array}\\ \text{ If x}>{\text{EC}}_{\text{c}\text{r}}\\ \begin{array}{c} \;\;\;\;\;\;\;\;\;\;\;\;\;\;\;\;\;\;\;\;\;\;\;\;\;\;\;y=a_{2}x+\beta_{2} \end{array} \end{array} \end{array}\}$$

where EC_cr_, the salinity level at the break point of the piecewise linear; x, the soil salinity (dS m^−1^); α_1_, α_2_, the slope of the first and second linear regressions, respectively, and β_1_, β_2_ are the intercepts of the two phases, respectively.

The logistic function used three parameters as described in the following equation:

(2) $$y=\frac{Y_{max}}{1+e^{a\left(x-b\right)}}$$

where Y_max_, maximum value of the considered process; (*a)*, the slope at 50% reduction of the maximum value; and (*b)*, the critical salinity level value for a 50% reduction of y from Y_max_.

Two piecewise linear and logistic fitting equations for net leaf photosynthesis rate, transpiration rate and leaf conductance, developed from the greenhouse studies in Experiments 1 and 2, were validated with greenhouse data in Experiment 3 and field data collected at 42 DAS in Experiment 4. The goodness of fit for the regressions was estimated using the coefficient of determination (R^2^) and the normalized root mean square error (RMSE_n_) between observed and calculated values. The best fitting curve was defined by the lowest RMSE and RMSE_n_. Variability in salinity tolerance and resilience was evaluated among the three genotypes by comparing the two parameters (*a)*, the rate of decrease in the process considering a linear decrease to 50% loss; and (*b)*, the threshold salinity level causing 50% loss in the process from its maximum rate Y_max_.

## 3 Results

### 3.1 Developmental responses to salinity stress

**Fig. 1:**
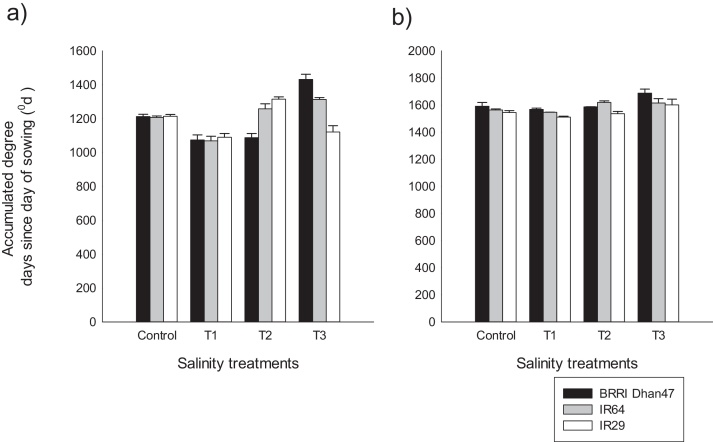
Panicle initiation (a) and flowering time (b) of the genotypes under different salinity treatments applied during the vegetative stage. Control: non-stressed conditions; T1, T2 and T3 are Treatment 1, 2 and 3 corresponding to salinity treatments at 4 dS m^−1^, 8 dS m^−1^, and 12 dS m^−1^, respectively. Values are average accumulated degree days (thermal time) from sowing, using 8 ^°^C as base temperature from observed values in Experiment 1 and 2 for 4–6 plants per treatment and per genotype.

The three genotypes had similar phenology with no significant variability among them. However, overall development of the plants was significantly affected by salinity treatment (Fig. 1, supplementary Table 2). On average, panicle initiation was six days earlier for plants stressed in Treatment 1 compared to the control, corresponding to an accumulated degree days from sowing to panicle initiation of about 1078 ± 9 ^0^d and 1210 ± 3 ^0^d, respectively. Flowering was earlier by about three days for Treatment 1 (1542 ± 23 ^0^d), but delayed by five days for plants stressed in Treatment 3 (1688 ± 38 ^0^d), compared to the control (1567 ± 19 ^0^d, Fig. 1).

### 3.2 Plant growth responses to salinity

#### 3.2.1 Variability in plant growth at the end of the salinity treatments

**Fig. 2:**
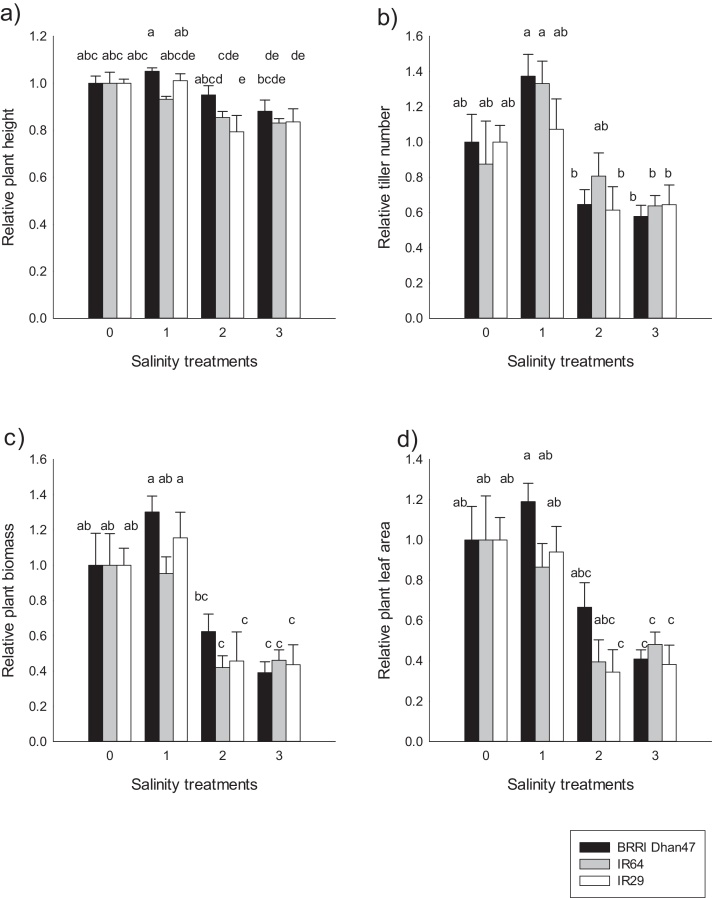
Plant growth response of the three genotypes at the end of the salt stress under different salinity treatments. a) relative plant height, b) relative plant tiller, c) relative plant biomass, d) relative plant leaf area. Control: non-stressed conditions; T1, T2 and T3: salinity treatments at 4 dS m^−1^, 8 dS m^−1^, and 12 dS m^−1^ in Experiments 1 and 2. Values are means of treatments relative to the control. Vertical bars indicate ± se. Means ± se were computed from individual values of 4–6 plants per treatment and per genotype in Experiment 1, 2. Bars labelled with the same letters did not present significant differences within a group of each treatment at *P* < 0.001.

Plant growth decreased progressively with increasing salinity stress, from the mild stress level of 4 dS m^−1^, but at different rates of reduction for the three genotypes (Fig. 2). No significant differences were observed between the control and Treatment 1 (4 dS m^−1^) for IR64 and BRRI Dhan47, but the sensitive genotype IR29 showed a significant increase in its growth. This was observed for leaf area index and total biomass (Supplementary Table 3). Under Treatment 2 (8 dS m^−1^), plant growth decreased significantly as reflected by the substantial reduction in plant biomass of 0.51 relative to the control (Fig. 2c). The greater reduction was observed in leaf area with a mean relative value of 0.36. Plant height and tiller number were less affected, with relative values of 0.77 and 0.95, respectively (Fig. 2a & b). A more severe and significant decrease in plant growth was observed under Treatment 3 (12 dS m^−1^), with no significant differences among genotypes. The sensitive genotype IR29 had the lowest relative biomass, leaf area and tiller number (respectively, 0.19, 0.15 and 0.40), while the tolerant genotype BRRI Dhan47 had the highest relative values of 0.34, 0.25 and 0.57, respectively. The relative decrease in plant height among genotypes was not significant, with a mean relative value of 0.65 (Fig. 2a, Supplementary Table 3).

#### 3.2.2 Variability in plant growth at flowering

**Fig. 3:**
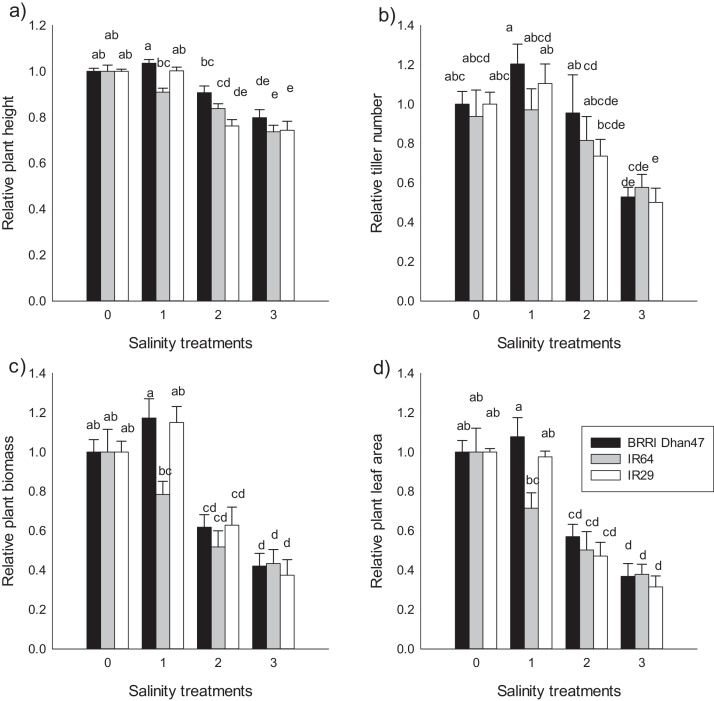
Relative responses in plant growth of the three genotypes at flowering stage under different salinity treatments. a) Relative plant height, b) Relative plant tiller, c) relative plant biomass, d) relative plant leaf area.Control: non-stressed conditions; T1, T2 and T3: salinity treatments at 4 dS m^−1^, 8 dS m^−1^, and 12 dS m^−1^ in Experiments 1 and 2. Values are means of the treatment relative to the control. Bars indicate ± se. Means ± se were computed from individual values of 4–6 plants per treatment and per genotype in Experiment 1, 2. Bars labelled with the same letters did not present significant difference within a group of treatment at *P* < 0.001.

After the withdrawal of the vegetative stage salinity treatment, the three genotypes recovered some of their growth by the time of flowering, particularly for plants subjected to Treatment 3 (12 dS m^−1^). For plant height, no significant differences were observed among salinity treatments and among genotypes (Fig. 3a). In contrast, significant decreases in tiller number, biomass, and leaf area were observed when plants subjected to increasing salinity were compared to the control (Fig. 3b–d). Treatment 2 (8 dS m^−1^), reduced average biomass to 0.71 of the control. IR29 showed the highest reduction, with a relative plant biomass of 0.59, which was lower than that for BRRI Dhan47 and IR64, at 0.77 and 0.76, respectively. Under Treatment 3 (12 dS m^−1^), the three genotypes showed a similar relative reduction in plant biomass with a decrease to 0.53 relative to the control (Fig. 3c).

Leaf area decreased to about 0.49 of the control under the high salinity treatment (Treatment 3) and to about 0.67 in Treatment 2. The highest decrease was observed for the sensitive genotype IR29, with a relative value of 0.46. Tiller number also declined significantly across genotypes to 0.67 under Treatment 3, relative to the control. However, no differences were observed between genotypes under Treatment 2 and 1 and those under control conditions (Fig. 3b). The highest reduction in tiller number was observed for BRRI Dhan47, with a relative value of 0.63. An increase of the tiller number was observed at flowering time compared to that after the stress period, suggesting the production of new tillers after the stress period.

### 3.3 Leaf gas exchange responses to salinity

Significant differences between genotypes and salinity treatments were observed at each date of measurement, with a significant interaction between genotype and salinity treatment for leaf net photosynthesis rate, transpiration rate and leaf conductance (Table 3). Differences due to leaf position were not significant except for IR29, where a significant difference was observed between younger and older leaves (Table 3). Interaction of salinity treatment with genotype was stronger (*P <* 0.001) than with experiment (*P <* 0.01).

**Table 3:** Variance analysis for each of the three leaf gas exchange variables among genotypes (G) and salinity treatments (S) in Experiments (Expt) 1–3. MSE refers to mean square error. Variables were measured at three times (28, 35 and 42 DAS, which were 7, 14 and 28 d after salinity treatment, respectively) on 4–6 plants per treatment and per genotype in Experiment 1, 2, and 3.

Factors	Net photosynthesis rate (μmol CO_2 _m^−2^ s^−1^)	Transpiration rate (mmol H_2_o m^−2^ s^−1^)	Leaf conductance (g H_2_o m^−2^ s^−1^)
	MSE	MSE	MSE
Genotype (G)	781.68***	41.07***	0.46***
Salinity (S)	1335.31***	183.75***	2.03***
Experiment	185.27**	93.05***	0.97***
Date of measurement (DAS)	1201.31***	28.7**	0.18**
Leaf position	86.43*	0.943 ns	0.08 ns
G × S	104.87**	15.5**	0.14***
S × Expt	131.77**	12.7*	0.06**
Errors	30.88	4.96	0.03

* P < 0.05.

** P < 0.01.

*** P < 0.001.

#### 3.3.1 Leaf net photosynthesis rate

**Fig. 4:**
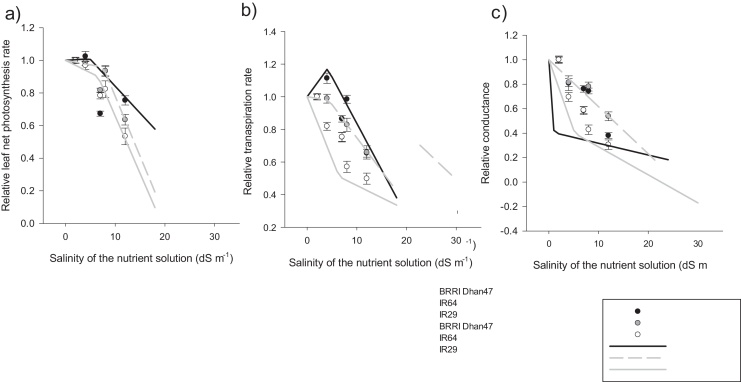
Leaf gas exchange variables and two piecewise linear function fitting the relative responses to salinity, a) Net photosynthesis, b) Transpiration and c) Conductance. Each point represents the mean value of the 3 experiments and the two dates of measurement for Experiments 1, 2 and 3 and field experiment (Experiment 4). Error bars represent variability across replicates (4–6 plants), experiments (Experiment 1 and 2, Experiment 3 and Experiment 4) and time of measurements (7, 14, 21 d after start of salinity treatment), together with ± standard error. The lines were fitted using linear regressions of the response patterns for each genotype.

**Fig. 5:**
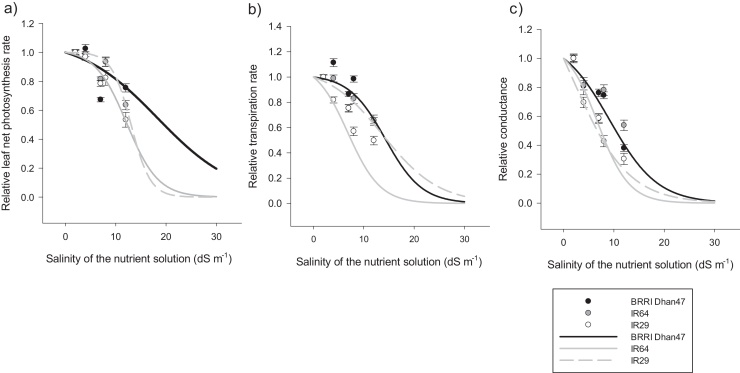
Leaf gas exchange variables and the logistic function fitting their relative responses to salinity, a) Net photosynthesis, b) Transpiration and c) Conductance. Each point represents the mean value of three experiments and two dates of measurement for Experiments 1, 2 and 3 and field experiment 4. Error bars represent variability across replicates (4–6 plants), experiments (Experiment 1 and 2, Experiment 3 and Experiment 4) and time of measurements (7, 14, 21 d after start of salinity treatment), together with ± standard error. The lines were fitted curves to response patterns for each genotype.

The effects of salinity and experiment were significant on net leaf photosynthesis rate (Table 3). Variability among genotypes was significant, with variance representing 20% of the total variation (Table 3). Net leaf photosynthesis rate varied from the highest at 39.1 μmol CO_2_ m^2^ s^−1^ for BRRI Dhan47 under control conditions in Experiment 3, to the lowest at 7.4 μmol CO_2_ m^2^ s^−1^ for IR29 measured 14 d after stress imposition under Treatment 3 in Experiment 2 (Supplementary Table 4). No differences were observed between genotypes under control and Treatment 1. However a significant decrease in net leaf photosynthesis was observed under Treatments 2 and 3 compared to the control (Fig. 4, Fig. 5).

#### 3.3.2 Leaf transpiration rate

Effects of salinity and experiment were significant on leaf transpiration rate (Table 3). Variability among genotypes was also significant, with variance representing 50% of the total variation in leaf transpiration (Table 3). However, under control conditions across all experiments and for all times of measurement, IR29 had the highest transpiration rate of 8.5 mmol H_2_O m^2^ s^−**1**^, which was significantly higher than the mean values for IR64 (7.08 mmol H_2_O m^2^ s^−1^) and BRRI Dhan47 (6.47 mmol H_2_O m^2^ s^−1^) (Y_max_, Table 5).

**Table 4:** Parameters for the two piece-wise linear function fitted for each of the three-leaf gas exchange variables in response to salinity as indicated in equation 1.

Genotype	Net photosynthesis rate (μmol CO_2_ m^−2^ s^−1^)					Transpiration rate (mmol H_2_o m^−2^ s^−1^)					Conductance (g H_2_o m^−2^ s^−1^)				
	Ec_cr_	_α_1	_α_2	R^2^	RMSEn (%)	Ec_cr_	_α_1	_α_2	R^2^	RMSEn (%)	Ec_cr_	_α_1	_α_2	R^2^	RMSEn (%)
BRRI Dhan47	4.82	0.04	−0.71	0.96	7.0–10.7	4.057	0.25	−0.34	0.91	7.0–33.4	1.04	−0.6	−0.01	0.93	26.0–39.2
IR64	7.58	−0.11	−1.45	0.99	3.0–17.7	4.26	−0.04	−0.29	0.99	1.0–15.7	–	−0.04	–	0.89	9
IR29	6.54	−0.32	−1.5	1	0.0–39.5	6.427	−0.76	−0.15	0.99	0.0–16.4	5.19	−0.096	−0.019	0.95	1.0–54.5

EC_cr_, critical level of salinity (dS m^−1^) to cause change in response; α_1_, α_2_, slopes of the two-piecewise linear regression; R^2^, coefficient of correlation; and RMSEn, normalized root mean square error between the observed and calculated values from the greenhouse experiments (Experiments 1, 2 and 3) and the field experiment (Experiment 4). Fitted data were obtained from 4 to 6 plants in each experiment (1–4) for each genotype and each Treatment (control, 1, 2 and 3) measured at three times (7, 14 and 21 d after start of salinity treatment).

**Table 5:** Parameters for the logistic function fitted for each of the three leaf gas exchange variables in response to salinity as indicated in equation 2.

Genotype	Net photosynthesis rate					Transpiration rate					Conductance				
	Y_max_ (μmol CO_2_ m^−2^ s^−1^)	*B*	*A*	R^2^	RMSEn (%)	Y_max_ (mmol H_2_o m^−2^ s^−1^)	*B*	*a*	R^2^	RMSEn (%)	Y_max_ (g H_2_o m^−2^ s^−1^)	*b*	*a*	R^2^	RMSEn (%)
BRRI Dhan47	25.46 ± 1.08	16.15 ± 0.4	0.19 ± 0.0	0.88	11.8–9.2	6.41 ± 0.24	14.30 ± 0.3	0.21 ± 0.0	0.72	17.9–27.6	0.47 ± 0.03	11.68 ± 0.6	0.34 ± 0.1	0.92	15.5–8.6
IR64	19.47 ± 0.9	12.39 ± 1.3	0.26 ± 0.1	0.61	23.7–17.5	7.08 ± 0.43	10.09 ± 0.7	0.21 ± 0.0	0.54	20.0–11.1	0.47 ± 0.04	8.92 ± 0.3	0.26 ± 0.0	0.72	24.6–17.6
IR29	20.61 ± 0.89	11.08 ± 1.4	0.31 ± 0.1	0.59	19.9–31.9	8.52 ± 0.42	6.83 ± 0.2	0.31 ± 0.1	0.38	32.1–3.8	0.63 ± 0.03	6.77 ± 0.5	0.32 ± 0.0	0.41	23.2–7.9

Y_max_, maximum value for the rate of the process under control conditions; *b*, level of salinity at 50% reduction (dS m^−1^); *a* is the slope of the linear regression at 50% reduction; R^2^, coefficient of correlation; RMSE_n_, normalized root mean square error between the observed and calculated values from the greenhouse experiments (Experiments 1, 2 and 3) and field experiment (Experiment 4). Fitted data were obtained from 4 to 6 plants in each experiment (1–4) for each genotype and each Treatment (control, 1, 2 and 3) measured at three times (7, 14 and 21 d after start of the salinity treatment). Values are means ± se.

Leaf transpiration rates under Treatments 1 and 2 were statistically similar to that of the control for IR64 and BRRI Dhan47. Under Treatment 3, transpiration rate decreased significantly to the relative values of 0.53 and 0.65 for IR29 and IR64, respectively, but with no significant differences among genotypes. (Figs. 4 b and 5 b).

#### 3.3.3 Leaf conductance

Leaf conductance showed high correlation with transpiration (R^2^ = 0.86, *P <* 0.001) and was significantly affected by treatment, genotype and experiment (Table 3). Leaf conductance ranged from 0.90 to 0.07 g H_2_O m^2^ s^−1^, respectively, for IR29 in Experiment 3 (control, 7 d after treatment) and IR64 in Experiment 1 (14 d after start of Treatment 3). At a salinity level of 12 dS m^−1^, no significant difference was observed between IR29 and BRRI dhan47, with relative reduction values of 0.36 and 0.40, respectively.

### 3.4 Genotypic variability in the pattern of leaf gas exchange responses to salinity

The three genotypes differed significantly in the level of salinity at which they become responsive. BRRI Dhan47 showed the highest level of salinity at which plant responses changed from a first linear phase to a second linear one (from 11.68 ds m^−1^), while IR29 presented the lowest levels (from 6.77 ds m^−1^) (Table 4). The rate of decrease in leaf gas exchange beyond these salinity levels also varied significantly, with BRRI Dhan47 having a lower rate than the sensitive genotype IR29.

The piecewise linear function used to fit these responses showed different trends among the processes and among genotypes (Table 4). The first phase of decrease was fitted with a slope α_1_ ranging from −0.04 to −0.76, suggesting an apparent growth maintenance to a very sensitive response until the breaking point (EC_cr_) before the second phase. Therefore, the breaking point was not always preceded by growth maintenance but was consistently followed by a further decrease (slope α_2_ = −0.01 to −1.5) (Fig. 4).

The tolerant genotype BRRI Dhan47 had the lowest breaking point (3.35 dS m^−1^), indicating that the genotype adjusted its function at lower levels of stress (Table 4). Its leaf conductance decreased at a faster rate (about −0.44) during the first phase (Fig. 4c). However, net photosynthesis and transpiration rates were relatively maintained (Figs. 4a and 2b). Reduction in transpiration and net photosynthesis were more severe during the second phase, with the slopes (α_2_) ranging from −0.35 to −0.71, respectively (Table 4).

The sensitive genotype IR29 showed the lowest decrease (α_1_) in conductance (−0.10), but higher reduction in transpiration (−0.76) and net photosynthesis (−0.11). The breaking point (EC_cr_) between the two linear phases occurred at 6.05 dS m^−1^ for leaf net photosynthesis of IR29, the highest value among the genotypes (Table 4). During the second phase, α_2_ was lower for leaf conductance (-0.01) and higher for leaf transpiration and net photosynthesis of −0.29 and −1.5, respectively.

As expected, the intermediate genotype IR64 showed an intermediate response between the tolerant and sensitive genotypes, with an average EC_cr_ of 5.92 dS m^−1^. During the first phase, its transpiration rate was relatively maintained (-0.04) and its net photosynthesis rate declined (α_1_ = −0.11). In the second phase, both processes drastically declined with α_2_ values of −0.29 and −1.47, respectively (Fig. 4a and b).

For the logistic function, a good fit with an R^2^ value ranging from 0.38 to 0.92 was observed. Parameters characterizing the fitting equations among genotypes were significantly different in their maximum, while the genotype BRRI Dhan47 showed the highest (*b)* value at which 50% reduction in maximum value occurs. Leaf conductance and net photosynthesis decreased to 0.5 of the control at salinity level ≥ 12 dS m^−1^ (11.68 dS m^−1^ and 16.15 dS m^−1^, respectively (Table 5)). For the genotype IR29, leaf conductance and transpiration rate decreased to 50% at a lower salinity level with (*b)* values <7 dS m^−1^ (6.77 dS m^−1^ and 6.83 dS m^−1^, respectively). Furthermore, the *(b)* value for IR29 leaf net photosynthesis was below 12 dS m^−1^ (11.08 dS m^−1^, Table 5). IR64 showed similar sensitivity as IR29, with *b* values for transpiration and net photosynthesis at 7.08 dS m^−1^ and 12.38 dS m^−1^, respectively.

For the slope (*a)*, defined as the rate at which the process is decreasing, BRRI Dhan47 showed the smallest slope of 0.19 for net photosynthesis, which was significantly lower than both IR29 and IR64 at 0.31 and 0.26, respectively (Table 4 and Fig. 5a). For leaf transpiration rate, IR29 had an *(a)* value of 0.31, which was significantly higher than that of BRRI Dhan47 and IR64. In contrast for leaf conductance, the parameter *(a)* did not differ significantly among genotypes (Fig. 5c).

## 4 Discussion

### 4.1 Variability in rice growth responses to salinity

Significant differences in plant growth and leaf gas exchange variables were observed between the control and the three different salinity treatments during vegetative stage in both the greenhouse and field experiments. At the end of the stress period, plant biomass was reduced to between 0.36 and 0.61 in Treatment 2 (8 dS m^−1^) relative to the control. This result is consistent with the reported rice crop responses at this level of salinity. For instance, grain yield was reported to decrease to 0.5 at a mean seasonal soil salinity of 6.77 dS m^−1^ (Grattan et al., 2002). Similar decreases in plant biomass have been reported for the genotype IR64 and IR29 under salinity stress of 6 dS m^−1^ (Castillo et al., 2004, Moradi and Ismail, 2007).

IR29 was most affected under the highest salinity treatment of 12 dS m^−1^, where its biomass decreased to 0.19 of the control at the end of the treatment. The genotype BRRI Dhan47 consistently responded as a salt-tolerant genotype, with its biomass reduced by only 25% at the end of the stress and by 50% at flowering. It is likely that under higher salinity conditions (≥12 dS m^−1^), the severe osmotic stress limiting plant growth and function was equivalent to an apparent water stress of 516 kPa in Experiments 1 & 2 (Supplementary Table 1) with Treatment 3 suggesting that at higher levels the two component effects of salinity (osmotic stress and ion toxicity) were over-shadowed by the severe stress conditions due to limited water uptake. Previous studies have reported similar ranges for decrease in rice growth under similar water deficits (Wopereis et al., 1996, Castillo et al., 2007).

Significant interaction between genotype and salinity treatment was observed. This interaction was observed for the plants under mild salinity stress treatment (4 dS m^−1^). No significant difference in plant growth was observed between BRRI Dhan 47 and IR64 under stress compared to the control, but a significant increase in plant biomass and leaf area index was observed in IR29 under stress. Rice was reported to be unaffected by salt stress at this lower salinity after the 5- leaf stage (Gregorio et al., 2002). As the salt stress in the current experiment was initiated at 21 DAS, the seedlings were already beyond the critically sensitive stage and were expected to tolerate mild stress (below 4 dS m^−1^). An increase in plant growth was even observed for both IR29 and BRRI Dhan47, suggesting stimulation of growth. This increase in growth under mild salinity has been attributed to an increase in root uptake of nutrients, mainly driven by the difference in osmotic potential between the plant and the soil solution (Marcelis and van Hooijdonk, 1999). Passive uptake of soil nutrients was reported at a xylem osmotic potential of 200 kPa. The osmotic potential of a soil solution at 4 dS m^−1^ is lower than that level (Horie et al., 2012, Schröder et al., 2014). Defining this critical level of water salinity is important for management of rice field irrigation with brackish water, particularly in determining amount and timing of saline water to be mixed with fresh water when the latter is scarce.

Based on the variability in plant growth responses to increasing salinity from one level to another, the rice plant biomass decrease was fitted using a three-step linear regression (Supplementary Fig. 3). A first linear phase characterized by a plateau takes place before the critical level of salinity (significant decrease point) occurs. A second phase then follows where the plant responds to the osmotic stress of the saline soil solution and results in a slowdown in its function and is likely limiting uptake of toxic ions such as Na^+^. A third phase occurs when a critical amount of sodium has been accumulated. In this condition, the plant’s system is affected by ion toxicity resulting in an irreversible destruction of functional cells and tissues. This new description is consistent with the two-phase response of wheat plants to salinity described by Munns et al. (1995). Based on the extent of tolerance of a genotype, the first phase was broken down into two phases allowing the determination of the level of tolerance within and between species. This further integrates the dimension of the duration of exposure to the stress while expressing the response based on accumulated Na^+^. No genotypic differences in plant growth were observed at the highest salinity level (12 dS m^−1^, Treatment 3), suggesting that water uptake was the dominant factor in that treatment. Even the tolerant genotype was overwhelmed due to both osmotic stress and higher accumulation of salt in plant tissues resulting in dual effects of toxicity and internal dehydration.

### 4.2 Modelling leaf gas exchange responses to salinity

The trends of leaf gas exchange response to salinity have been fitted with both a two piecewise linear function and a logistic function determined by three parameters. The two piecewise linear function achieved a higher coefficient of determination than the logistic function as it captured specific responses of the crop growth related to the current experimental conditions. The shape of the two piece-wise functions was not consistent among genotypes or among variables. The individually linear parameters were not comparable among genotypes and indicated a more environmental (experiment and time of measurement) factor dependence. For instance in Experiment 2, salinity had less effect with the dilution effect of high biomass production, mitigating the stress impact. Plants showed higher biomass production and thus higher transpiration and photosynthesis as they were growing in the dry season with higher radiation. In contrast, plants in Experiment 1 showed lower biomass production, lower transpiration and leaf net photosynthesis, resulting in less dilution of accumulated salt, thus causing early trigger of responses to salinity. It is likely that the two piecewise linear function captured the plasticity of responses to salinity in these rice genotypes due to stimulation and slowing of processes, as indicated by the variability of the linear coefficients (Table 4, Fig. 4). The replicability of the function is then limited, as was observed for the leaf conductance where one linear regression would result in a better fit than a two piecewise linear function (Fig. 4 c).

In addition, the RMSE_n_ of the two piecewise linear function was higher compared to the RMSE_n_ of the logistic function, based on the observed data from the field experiment (Table 5), suggesting that the sigmoid function presented lower error and thus better fit than the two piecewise linear function.

The trends of the genotypic responses were similar and consistent with their level of tolerance when a logistic function was used. A sensitive cultivar was defined as having the lowest threshold value of salinity that reduced the leaf gas exchange variables to 50% of their value under the control treatment and having the highest reduction rate value. In contrast, a tolerant cultivar like BRRI Dhan 47 had the highest value of (*b)* and the lowest value of *(a)*. These parameters are therefore useful indicators for salinity tolerance and can be of interest for phenotyping, as they are measurable and indicative of genotypic variability.

It is worth noting that the severity of salinity impact on the rice crop is greater in drier environments (Asch and Wopereis, 2001). Thus, the salinity levels we defined as thresholds to cause a significant effect on plant functions are limited to the environmental conditions of the experiments, assuming that toxic salt accumulation is driven by transpiration. Future validation of these values under drier environments is desirable to confirm the tolerance level of the genotypes. However, relative genotypic difference may remain similar under low humidity since the tolerant genotype, BRRI Dhan47, can adjust its transpiration rate at a lower salinity levels than IR29 and IR64, both of which maintained their transpiration up to a salinity level of 8 dS m^−1^. Since BRRI Dhan 47 has been reported to tolerate salinity stress above 12 dS m^−1^ (Islam et al., 2008), a study on the performance of BRRI Dhan47 under drought stress could confirm if the delayed responses of its transpiration at higher salinity levels are mainly driven by osmotic stress or by salt exclusion ability. Studies of tissue tolerance would also confirm if the ability of BRRI Dhan 47 to maintain photosynthesis is due to higher ion toxicity tolerance involving compartmentation within cells or tissues as reported for other tolerant rice genotypes (Flowers et al., 1985, Yeo et al., 1988).

The parameters defined for leaf gas exchange responses correlated with plant growth responses to salinity and could be of interest to identify traits that are critical for tolerance. Integrating the logistic equations into crop models could link the process responses directly to crop yield assessment, in which the parameter *(b)* will quantify the tolerance of the genotype and the parameter *(a)* its resilience. The logistic function further allows representation of growth maintenance or decrease with temporal variability of soil salinity compared with the usual seasonal salinity effect representation in most crop models. Early reports in modelling rice crop responses to salinity in the indica subgroup have reported a threshold of salt stress for plant biomass and yield reduction at 3 dS m^−1^, with a decrease of 13% per unit increase in salinity beyond this threshold (Maas and Hoffman, 1977, Maas et al., 1999). This critical value reflects variability among genotypes when complex plant processes contributing to yield, such as photosynthesis and its interactions with limiting environmental factors, such as salinity, were considered. With the recently released salt-tolerant varieties, such as BRRI Dhan47, this threshold has increased to more than 4 or 5 dS m^−1^ and the rate of decrease in *(a)* declined to as low as 4% per unit of salinity increase beyond the threshold. The impact of this change in threshold on yield would be interesting to quantify. Variation in salinity tolerance in rice and crop responses to salinity during the reproductive stage have also been reported as independent of the mechanisms involved in tolerance during vegetative stage (Moradi et al., 2003, Ahmadizadeh et al., 2016). Similar investigations would, therefore, be of interest for the entire crop growth duration to better capture the contribution and impact of tolerance on the crop yield variability in saline fields. The capacity of the genotype to recover after the salinity treatment also suggests the need for a complex approach in quantifying the salinity effect. For instance, the production of new tillers after the stress may result in yield loss reduction if these tillers were productive. Additional work is on-going to explore the integration of these findings into the rice crop model ORYZA (Bouman et al., 2001, Li et al., 2017) to assess the effects of the observed variability at the whole plant level for genotypes with different tolerance thresholds. This offers a mechanistic description of the genotypic variability of rice crop responses to salinity, while limiting the number of parameters for the model. This type of quantification can also be performed for additional new varieties using crop modelling (Karlberg et al., 2006), to estimate expected gains and to determine the geographical regions where these varieties will likely be more successful.

## 5 Conclusions

Several salt-tolerant rice varieties were recently released with the potential to increase and sustain rice productivity in salt-affected areas. This study developed approaches to characterize and quantify tolerance in these varieties. At a high salinity level of 12 dS m^−1^, no significant differences in biomass were observed among the contrasting rice genotypes evaluated in this study, indicating that even the tolerant genotype was overwhelmed due to both osmotic stress and higher accumulation of salt in plant tissues, causing both toxicity and internal dehydration. The responses of leaf gas exchange variables were fitted using both piecewise linear and logistic functions. The logistic equation allowed better comparison among genotypes and provided a simplistic quantification approach for their tolerance, providing parameters that quantified both tolerance and resilience of a genotype. For the tolerant genotype BRRI Dhan47, a 50% loss in leaf conductance did not occur until a salinity level of 11.08 dS m^−1^, but BRRI Dhan47adjusted its stomatal conductance at a lower level than the other genotypes (1.04 dS m^−1^). Although the transpiration and net photosynthesis rates of BRRI Dhan47 decreased to 50% only at salinity levels of 14.30 and 16.15 dS m^−1^, respectively, the slope of this reduction was statistically similar to that of IR64 and IR29, thereby suggesting a window for further improvement in this trait to future enhance salinity tolerance. For future studies, we propose an integration of these equations into crop modelling to evaluate the impact of the observed variability on rice yield. This might further contribute to improving modelling of rice production in salt-affected areas for both agronomic research and for virtual multi-location evaluation of breeding lines. Further investigations of these parameters using larger numbers of contrasting genotypes and genetic populations would be important, and could also facilitate further understanding of the traits associated with salinity tolerance and their genetic control during the vegetative stage. For whole-season crop responses, similar studies would be needed to understand and quantify genotypic variability in salinity tolerance during the reproductive stage. Since different important mechanisms are involved during these two phases, quantifying the salinity effect on kernel formation from initiation through pollination and grain filling would be necessary to define variability in grain size and number, and effects on the source capacity caused by leaf senescence, and biomass partitioning. Linking this quantification approach with crop modelling, trait-based selection and genome-wide association would provide innovatively integrated approaches for rice breeding.
