# Supplementary material for: Describing the physiological responses of different rice genotypes to salt stress using sigmoid and piecewise linear functions
Source: Field Crops Res. 2018 May 1;220:46–56. doi: 10.1016/j.fcr.2017.05.001 (PMC5890386; doi:10.1016/j.fcr.2017.05.001)
Supplement: Supplementary file 1 [file mmc1.docx]

| s |
| --- |

Supplementary Figure 1. An Example of nutrient solution electrical conductivity dynamics for Experiment 2. Each line represents dynamic average daily values of soil electrical conductivity measured every 30 min at 15 cm depth from 3 pots in a tank with respective salinity treatment.

Supplementary Figure 2. Salinity of soil solution in Experiment 4. Data are averages of continuous measurements in three replications. Each line represents dynamic average daily values of soil electrical conductivity measured every 30 min at 15 cm depth from 3 replicates (blocks) under control and salinity treatments.

Phase 1

Phase 2

Phase 3

Supplementary Figure 3. Variation in plant biomass (g plant ^-1^) with salinity treatments. The black line represents a mean trend over experiments and genotypes of the three-step linear response to salinity in rice plant growth. Each point represents a mean of four to six plants per genotype in Experiments 1, 2 and 3 for each sampling date during the salinity treatment period.

Supplementary Table 1. Electrical conductivity (EC), osmotic potential, and NaCl concentration of the nutrient solution for each of the four salinity treatments. Values are means of four samplings of the nutrients solutions from each tank under respective salinity treatment.

| **Salinity (EC)** | Salt added | NaCl | EC | Osmotic Potential |
| --- | --- | --- | --- | --- |
|  | g l^-1^ | mM | dS m^-1^ | kPa |
| Control | - | - | 2.3 | 215 |
| Treatment 1 | 1.3 | 20 | 4.2 | 254 |
| Treatment 2 | 3.3 | 55 | 8.1 | 344 |
| Treatment 3 | 5.3 | 90 | 12.1 | 516 |
|  |  |  |  |  |

Supplementary Table 2. Variance analysis for panicle initiation and flowering time among genotypes (G) and salinity treatments (S) in Experiments (Expt) 1 to 2. MSE refers to mean square error. Variables were averaged from 4 to 6 plants per treatment and per genotype in Experiment 1 and 2.

| Factors | Panicle initiation  (μmol CO_2_ m^2^ s^-1^) | Flowering time  (mmol H_2_o m^2^ s^-1^) |  |
| --- | --- | --- | --- |
|  | MSE | MSE |  |
| Genotype (G) | 5112ns | 7458* |  |
| Salinity (S) | 103219*** | 9180* |  |
| G x S | 62199** | 1071 |  |
| Errors | 13823 | 1763 |  |
| *, **, *** represents significance at P < 0.05, 0.01, and 0.001, respectively | | |  |

Supplementary Table 3. Plant growth variables under different salinity treatments at the end of the stress treatments and at flowering. Values are means of 3 to 6 plants from each experiment with ± standard deviation for each treatment (Treatment 1 and 2). Values with different letters within a column are significantly different at *P* < 0.001.

| **Treatment** | **Genotype** | **End of the salinity treatment** | | | | **Flowering time** | | | |
| --- | --- | --- | --- | --- | --- | --- | --- | --- | --- |
|  |  | **Plant biomass**  **(kg ha^-1^)** | **Leaf area index**  **(m^2^ m^-2^)** | **Tiller**  **(number pl^-1^)** | **Plant Height**  **(cm)** | **Plant biomass**  **(kg ha^-1^)** | **Leaf area index**  **(m^2^ m^-2^)** | **Tiller**  **(number pl^-1^)** | **Plant Height**  **(cm)** |
| Control | BRRI Dhan47 | 1580±259^b^ | 2.36±0.55^c^ | 6.14±2.25^cd^ | 81.6±8.7^a^ | 20876±14977^b^ | 11.54±6.19^ab^ | 21.75±11.89^ab^ | 129.8±11.0^ab^ |
|  | IR29 | 1693±6496^b^ | 2.94±0.74^b^ | 8.36±2.46^bc^ | 75.7±6.7^abc^ | 16663±5959^bc^ | 10.24±3.46^abc^ | 28.89±8.39^a^ | 109.2±6.6^cd^ |
|  | IR64 | 2204±744^a^ | 3.13±0.27^ab^ | 10.0±3.10^b^ | 78.3±5.0^ab^ | 18556±12866^b^ | 9.63±6.54^abcd^ | 22.57±14.79^ab^ | 118.2±15.6^bc^ |
| Treatment 1  4 dSm^-1^ | BRRI Dhan47 | 1585±677^b^ | 2.41±1.08^c^ | 8.20±4.92^bc^ | 73.0±10.0^bc^ | 29146±10549^a^ | 14.60±4.48^a^ | 30.88±7.86^a^ | 136.5±5.6^a^ |
|  | IR29 | 2576±481^a^ | 3.61±0.74^a^ | 13.43±7.39^a^ | 73.8±5.2^abc^ | 19829±9566^b^ | 9.67±4.04^abcd^ | 31.22±15.73^a^ | 110.4±10.0^cd^ |
|  | IR64 | 1448±312^b^ | 2.12±0.56^c^ | 8.33±4.50^bc^ | 67.5±4.7^c^ | 19312±8361^b^ | 8.70±3.79^bcd^ | 30.25±8.65^a^ | 110.2±4.1^cd^ |
| Treatment 2  8 dSm^-1^ | BRRI Dhan47 | 718±231^c^ | 0.85±0.29^d^ | 4.64±2.46^d^ | 68.0±9.5^c^ | 12757±8263^bc^ | 7.42±3.26^bcd^ | 13.14±5.11^b^ | 123.4±13.8^abc^ |
|  | IR29 | 918±462^c^ | 0.94±0.52^d^ | 6.00±4.24^cd^ | 55.7±3.3^d^ | 8361±7789^c^ | 3.57±2.84^d^ | 18.00±9.88^ab^ | 87.0±20.7^e^ |
|  | IR64 | 762±381^c^ | 0.90±0.49^d^ | 6.17±4.96^cd^ | 57.0±6.8^d^ | 7790±4829^c^ | 3.91±3.15^d^ | 18.14±8.41^ab^ | 101.0±8.1^de^ |
| Treatment 3  12 dSm^-1^ | BRRI Dhan47 | 638±227^c^ | 0.70±0.31^d^ | 3.67±1.80^d^ | 54.4±8.7^d^ | 7575±3559^c^ | 4.55±1.25^cd^ | 11.71±3.20^b^ | 114.4±16.5^cd^ |
|  | IR29 | 384±151^c^ | 0.47±0.24^d^ | 3.29±2.06^d^ | 46.4±4.9^e^ | 6714±3652^c^ | 3.88±2.30^d^ | 18.50±7.48^ab^ | 90.9±12.8^e^ |
|  | IR64 | 431±290^c^ | 0.53±0.43^d^ | 4.17±3.87^d^ | 46.9±11.4^e^ | 8361±3545^c^ | 4.62±1.81^cd^ | 14.25±3.62^b^ | 98.2±6.3^de^ |

Supplementary Table 4. Leaf net photosynthesis (μmol CO_2_ m^-2^ s^-1^) under different salinity treatments in Experiments 1, 2, 3 and 4. Values are means of 3 to 6 plants from each experiment at three times of measurement (28, 35 and 42 d after sowing) in Experiments 1, 2 and 3 and at 42 d after sowing in Experiment 4. Data are means ± SD. Values with different letters within a column are significantly different at *P* < 0.001.

| **Treatment** | **Genotype** | **Experiment 1** | | | | **Experiment 2** | | | | **Experiment3** | | | | **Experiment4** | | |
| --- | --- | --- | --- | --- | --- | --- | --- | --- | --- | --- | --- | --- | --- | --- | --- | --- |
| Control | BRRI Dhan47 | 23.32 | ±6.06 | _a_ | 30.69 | | ±10.75 | _a_ | 22.32 | | ±4.63 | _a_ | 24.38 | | ±0.26 | _a_ |
|  | IR64 | 20.52 | ±6.45 | _abc_ | 18.58 | | ±4.78 | _cd_ |  | |  |  | 22.14 | | ±4.67 | _ab_ |
|  | IR29 | 19.35 | ±5.23 | _abc_ | 19.62 | | ±5.11 | _cd_ | 22.24 | | ±6.69 | _a_ | 20.18 | | ±3.47 | _ab_ |
| Treatment 1  4 dSm^-1^ | BRRI Dhan47 | 18.11 | ±6.41 | _abc_ | 24.32 | | ±5.59 | _b_ |  | |  |  |  | |  |  |
|  | IR64 | 19.36 | ±5.98 | _abc_ | 17.40 | | ±3.63 | _cd_ |  | |  |  |  | |  |  |
|  | IR29 | 22.29 | ±6.69 | _ab_ | 20.96 | | ±4.28 | _bc_ |  | |  |  |  | |  |  |
| Treatment 2  8 dSm^-1^ | BRRI Dhan47 | 21.26 | ±5.51 | _ab_ | 17.99 | | ±6.41 | _cd_ | 21.05 | | ±3.12 | _a_ |  | |  |  |
|  | IR64 | 17.31 | ±5.15 | _abc_ | 19.64 | | ±4.70 | _cd_ |  | |  |  |  | |  |  |
|  | IR29 | 14.37 | ±6.46 | _c_ | 17.69 | | ±5.83 | _cd_ | 21.27 | | ±7.03 | _a_ |  | |  |  |
| Treatment 3  12 dSm^-1^ | BRRI Dhan47 | 16.23 | ±3.34 | _bc_ | 16.36 | | ±5.28 | _cd_ |  | |  |  | 17.53 | | ±2.38 | _b_ |
|  | IR64 | 9.94 | ±3.45 | _d_ | 14.68 | | ±5.85 | _d_ |  | |  |  | 17.89 | | ±1.72 | _b_ |
|  | IR29 | 8.24 | ±3.95 | _d_ | 14.47 | | ±4.92 | _d_ |  | |  |  | 17.78 | | ±9.47 | _b_ |

Supplementary Table 5. Leaf transpiration rate (mmol H_2_o m^-2^ s^-1^) under different salinity treatments in Experiments 1, 2, 3 and 4. Values are means obtained from 3 to 6 plants from each experiment at three times of measurements (28, 35 and 42 d after sowing) in Experiments 1, 2 and 3 and at 42 d after sowing in Experiment 4. Data are means ± SD. Values with different letters within a column are significantly different at *P* < 0.001.

| **Treatment** | **Genotype** | **Experiment 1** | | | | **Experiment 2** | | | | **Experiment3** | | | | **Experiment4** | | |
| --- | --- | --- | --- | --- | --- | --- | --- | --- | --- | --- | --- | --- | --- | --- | --- | --- |
| Control | BRRI Dhan47 | 6.16 | ±2.07 | _abcd_ | 5.89 | | ±1.31 | _b_ | 7.10 | | ±1.98 | _b_ | 13.92 | | ±0.49 | _a_ |
|  | IR64 | 6.72 | ±2.95 | _abc_ | 7.39 | | ±2.24 | _ab_ |  | |  |  | 13.12 | | ±0.99 | _a_ |
|  | IR29 | 7.60 | ±2.79 | _ab_ | 7.39 | | ±2.11 | _ab_ | 10.01 | | ±3.40 | _a_ | 13.34 | | ±1.16 | _a_ |
| Treatment 1  4 dSm^-1^ | BRRI Dhan47 | 5.37 | ±2.63 | _abcd_ | 7.74 | | ±1.98 | _a_ |  | |  |  |  | |  |  |
|  | IR64 | 4.15 | ±2.55 | _cd_ | 7.39 | | ±1.77 | _ab_ |  | |  |  |  | |  |  |
|  | IR29 | 7.87 | ±2.85 | _a_ | 6.76 | | ±1.52 | _ab_ |  | |  |  |  | |  |  |
| Treatment 2  8 dSm^-1^ | BRRI Dhan47 | 5.15 | ±2.19 | _bcd_ | 6.15 | | ±2.44 | _ab_ | 6.14 | | ±1.03 | _b_ | 11.18 | | ±0.10 | _b_ |
|  | IR64 | 5.17 | ±2.21 | _bcd_ | 6.86 | | ±1.82 | _ab_ |  | |  |  | 9.75 | | ±1.46 | _c_ |
|  | IR29 | 4.80 | ±2.11 | _cd_ | 6.54 | | ±2.44 | _ab_ | 7.10 | | ±2.96 | _b_ | 9.56 | | ±2.57 | _c_ |
| Treatment 3  12 dSm^-1^ | BRRI Dhan47 | 3.64 | ±0.84 | _d_ | 4.25 | | ±1.25 | _c_ |  | |  |  | 11.18 | | ±0.10 | _b_ |
|  | IR64 | 4.56 | ±2.89 | _cd_ | 4.68 | | ±1.50 | _c_ |  | |  |  |  | |  |  |
|  | IR29 | 4.01 | ±1.76 | _cd_ | 4.66 | | ±1.79 | _c_ |  | |  |  |  | |  |  |

Supplementary Table 6. Leaf stomatal conductance (g H_2_o m^-2^ s^-1^) under different salinity treatments in Experiments 1, 2, 3 and 4. Values are means of 3 to 6 plants from each experiment at three times of measurement (28, 35 and 42 d after sowing) in Experiments 1, 2 and 3 and at 42 d after sowing in Experiment 4. Data is presented as means ± SD. Values with different letters within a column are significantly different at *P* < 0.001.

| **Treatment** | **Genotype** | **Experiment 1** | | | | **Experiment 2** | | | | **Experiment3** | | | | | | **Experiment4** | | |
| --- | --- | --- | --- | --- | --- | --- | --- | --- | --- | --- | --- | --- | --- | --- | --- | --- | --- | --- |
| Control | BRRI Dhan47 | 0.36 | ±0.20 | _ab_ | 0.42 | | ±0.16 | _bcd_ | 0.56 | | | ±0.20 | | _b_ | 1.56 | | ±0.01 | _a_ |
|  | IR64 | 0.42 | ±0.17 | _ab_ | 0.52 | | ±0.24 | _abc_ |  | | |  | |  | 1.32 | | ±0.16 | _b_ |
|  | IR29 | 0.51 | ±0.22 | _a_ | 0.60 | | ±0.29 | _a_ | 0.75 | | | ±0.20 | | _a_ | 1.39 | | ±0.28 | _ab_ |
| Treatment 1  4 dSm^-1^ | BRRI Dhan47 | 0.26 | ±0.12 | _bc_ | 0.57 | | ±0.23 | _ab_ |  | | |  | |  |  | |  |  |
|  | IR64 | 0.24 | ±0.12 | _bc_ | 0.43 | | ±0.16 | _bcd_ |  | | |  | |  |  | |  |  |
|  | IR29 | 0.50 | ±0.21 | _a_ | 0.48 | | ±0.19 | _abcd_ |  | | |  | |  |  | |  |  |
| Treatment 2  8 dSm^-1^ | BRRI Dhan47 | 0.27 | ±0.10 | _bc_ | 0.34 | | ±0.18 | _def_ | 0.35 | | | ±0.07 | | _c_ |  | |  |  |
|  | IR64 | 0.34 | ±0.21 | _ab_ | 0.39 | | ±0.14 | _cde_ |  | | |  | |  |  | |  |  |
|  | IR29 | 0.24 | ±0.12 | _bc_ | 0.44 | | ±0.23 | _bcd_ | 0.47 | | | ±0.18 | | _b_ |  | |  |  |
| Treatment 3  12 dSm^-1^ | BRRI Dhan47 | 0.13 | ±0.03 | _c_ | 0.20 | | ±0.07 | _f_ |  | | | |  |  | 0.98 | | ±0.17 | _c_ |
|  | IR64 | 0.27 | ±0.21 | _bc_ | 0.22 | | ±0.08 | _f_ |  | | | |  |  | 0.71 | | ±0.24 | _d_ |
|  | IR29 | 0.13 | ±0.07 | _c_ | 0.26 | | ±0.13 | _ef_ |  | |  | | |  | 0.68 | | ±0.31 | _d_ |
